# The Role of Circulating Tumor Cells as a Liquid Biopsy for Cancer: Advances, Biology, Technical Challenges, and Clinical Relevance

**DOI:** 10.3390/cancers16071377

**Published:** 2024-03-31

**Authors:** Tyler A. Allen

**Affiliations:** Duke Cancer Institute, Durham, NC 27710, USA; tyler.allen@duke.edu

**Keywords:** circulating tumor cells (CTCs), liquid biopsy, cancer metastasis, angiopellosis, cancer exodus hypothesis, microfluidic technologies, CTC detection and isolation, personalized oncology, metastatic disease monitoring, CTC clusters

## Abstract

**Simple Summary:**

This review article explores the forefront of research and technology in the detection and isolation of circulating tumor cells (CTCs) for liquid biopsy applications. Traditional biopsies, which necessitate tissue samples, are invasive and often challenging for patients. In contrast, liquid biopsies provide a less invasive alternative, enabling the continuous monitoring of the cancer’s progression and the effectiveness of treatments. Recent advancements have significantly enhanced our understanding of CTCs, underscoring their critical role in cancer research and patient management. This article aims to highlight these technological innovations and their potential to transform cancer diagnosis, prognosis, and personalized therapy, offering new hope for improved patient outcomes in the fight against cancer.

**Abstract:**

Cancer remains a leading cause of mortality worldwide, with metastasis significantly contributing to its lethality. The metastatic spread of tumor cells, primarily through the bloodstream, underscores the importance of circulating tumor cells (CTCs) in oncological research. As a critical component of liquid biopsies, CTCs offer a non-invasive and dynamic window into tumor biology, providing invaluable insights into cancer dissemination, disease progression, and response to treatment. This review article delves into the recent advancements in CTC research, highlighting their emerging role as a biomarker in various cancer types. We explore the latest technologies and methods for CTC isolation and detection, alongside novel approaches to characterizing their biology through genomics, transcriptomics, proteomics, and epigenetic profiling. Additionally, we examine the clinical implementation of these findings, assessing how CTCs are transforming the landscape of cancer diagnosis, prognosis, and management. By offering a comprehensive overview of current developments and potential future directions, this review underscores the significance of CTCs in enhancing our understanding of cancer and in shaping personalized therapeutic strategies, particularly for patients with metastatic disease.

## 1. Introduction

Cancer, a leading cause of mortality globally, presents a complex challenge in medical science, particularly due to its ability to metastasize (spread) to distant organs. The key to understanding and combating this metastatic spread lies in the study of circulating tumor cells (CTCs), which traverse the body’s vasculature, shedding light on the cancer’s progression and response to treatment [1]. The history of CTC research, dating back to the 19th century with the pioneering work of Australian physician Thomas Ashworth [2], has evolved dramatically, leading to a paradigm shift in cancer diagnostics and treatment strategies.

Before delving into the specifics of circulating tumor cells (CTCs), it is crucial to understand the broader context in which they operate within oncological research. Liquid biopsy, a minimally invasive technique, allows for the detection of cancer biomarkers directly from bodily fluids. Among these biomarkers, CTCs, cell-free DNA (cfDNA), and RNA provide a dynamic glimpse into the tumor’s genetic landscape. This revolutionary approach offers promising avenues for early cancer detection, monitoring disease progression, and tailoring personalized treatment strategies [3].

CTCs, once considered mere byproducts of the primary tumor, are now recognized as crucial players in the metastatic process. Their detection and analysis have paved the way for the development of liquid biopsy techniques, offering a minimally invasive alternative to traditional tissue biopsies. This innovative approach has revolutionized our understanding of tumor dynamics, enabling real-time monitoring and providing insights into tumor heterogeneity, treatment response, and disease progression [4,5,6,7,8].

An emerging area of interest in CTC research is the study of CTC clusters—groups of tumor cells bound together, traveling through the bloodstream. These clusters have been found to possess a higher metastatic potential compared to individual CTCs, offering new insights into the mechanisms of cancer spread and potential targets for therapeutic intervention [9,10,11,12,13].

Another intriguing aspect of circulating tumor cells (CTCs) in the bloodstream is their interaction with peripheral blood cells, such as monocytes and neutrophils. These interactions are not merely incidental but can significantly influence the metastatic potential and survival of CTCs. Recent studies suggest the possibility of hybrid cell formation and cell-in-cell phenomena during these interactions, reminiscent of mechanisms observed in primary tumor tissues [14,15]. Such cellular dynamics could offer new insights into the complexities of cancer metastasis and the role of the immune system in cancer progression, highlighting the importance of considering these interactions in the study of CTCs and liquid biopsies.

This review highlights several pivotal studies in the realm of CTC analysis, underscoring their collective contribution to our understanding of cancer metastasis and the development of liquid biopsy techniques. Each study, by elucidating the complex behavior and characteristics of CTCs, has incrementally advanced our ability to not only detect but also characterize these elusive cells with greater precision and sensitivity.

The advent of new technologies and methods for the isolation and detection of CTCs has been a cornerstone of recent advancements in oncology [16]. Techniques such as microfluidics, immunomagnetic separation, and advanced imaging have not only improved the sensitivity and specificity of CTC detection but have also opened new avenues for their characterization (Table 1). These technologies facilitate a deeper understanding of CTC biology, unraveling the complexities of their role in cancer dissemination [17].

Furthermore, the application of genomics, transcriptomics, proteomics, and epigenetic profiling to CTCs has provided a more comprehensive view of their molecular characteristics. This molecular profiling has significant implications for personalized medicine, allowing for the longitudinal identification of specific mutations and biomarkers that guide targeted therapy decisions over time [18,19,20].

The latest findings in CTC research have begun to make their way into clinical practice, transforming the management of various cancer types. From the early detection and monitoring of minimal residual disease to the assessment of therapeutic efficacy and the development of personalized treatment plans, CTCs are at the forefront of a new era in cancer care [6,7,21,22,23].

This review aims to provide an in-depth analysis of the advances in our understanding of the role of CTCs in cancer and liquid biopsy research, focusing on novel methodologies for their isolation and detection, the characterization of their biology, and the clinical implementation of these findings across different cancer types. By exploring the current state of knowledge and future directions, this review highlights the pivotal role of CTCs in the ongoing quest to understand and combat cancer and improve the efficacy of liquid biopsies for an improved clinical/patient outcome.

## 2. Review

### 2.1. Technological Advancements in CTC Detection and Isolation

A key component of using and researching CTCs involves being able to both detect and isolate them. Recent advancements in the imaging and isolation of CTCs have advanced the ability to both study and use them as key components of data in liquid biopsies.

#### 2.1.1. Microfluidic Platforms in CTC Isolation

The evolution of microfluidic platforms has significantly advanced the field of CTC isolation, marking a pivotal shift in liquid biopsy technologies. These platforms leverage the principles of microfluidics, which involve the manipulation of fluids at a sub-millimeter scale, to isolate CTCs from blood samples. These technologies have emerged as a crucial tool, offering a blend of precision, efficiency, and versatility (Table 2).

##### Development of Microfluidic Technologies for CTC Isolation

Microfluidic technologies have undergone substantial development over the past decade, driven by the need to overcome the challenges posed by the rarity and heterogeneity of CTCs in blood samples [24,25,26]. Traditional methods of CTC isolation often faced limitations due to the low frequency of these cells and their diverse phenotypic expressions. Microfluidic systems, with their intricate designs and precise fluid control, provide a solution to these challenges [7]. They enable the handling of small sample volumes and facilitate the integration of various functions, such as cell sorting and analysis, into a single compact device.

##### Application and Advantages

The application of microfluidic platforms in CTC isolation is multifaceted. These systems utilize the physical properties of CTCs, such as size and deformability, to distinguish them from other blood components [27]. This distinction is critical in ensuring the purity and integrity of isolated CTCs, which is essential for subsequent analyses. Microfluidic devices often employ techniques like microfiltration, where blood is passed through microfabricated channels or barriers that selectively capture CTCs while allowing other cells to pass through [28].

One of the key advantages of microfluidic platforms is their ability to preserve the viability of circulating tumor cells (CTCs) during isolation. This capability is pivotal for downstream applications, such as genomic and proteomic analyses, which necessitate intact cells for accurate and comprehensive analysis. Studies such as those by Bhat et al. (2022) and Stiefel et al. (2022) have demonstrated the effectiveness of microfluidic devices in maintaining CTC integrity, thereby facilitating detailed molecular characterizations post-isolation [29,30]. These analyses are crucial for understanding the metastatic potential of CTCs and for the development of targeted therapies. Furthermore, the high-throughput nature of these systems enables the processing of larger blood volumes, significantly enhancing the likelihood of capturing CTCs even in scenarios where they are present in low abundance [28,31,32]. This feature is particularly important for early cancer detection and the monitoring of minimal residual disease, where CTC numbers may be exceedingly sparse.

##### Clinical Implications and Future Prospects

The clinical implications of microfluidic-based CTC isolation are vast. These platforms have the potential to enhance cancer diagnosis, provide insights into tumor evolution, and aid in the monitoring of treatment responses [24,28]. As the technology continues to evolve, it is anticipated that microfluidic devices will become more integrated into clinical settings, offering a non-invasive, real-time window into the tumor biology of patients [28]. The work by Descamps et al. (2022) and Aghaamoo et al. (2023) exemplifies the rapid progress in microfluidic technologies for CTC isolation, showcasing how these platforms offer enhanced sensitivity and specificity in detecting these elusive cells, crucial for early cancer diagnosis and monitoring [24,25].

In conclusion, microfluidic platforms represent a significant advancement in the isolation of CTCs, contributing to the broader field of liquid biopsy. Their development and application underscore the ongoing efforts to refine cancer diagnostic tools, paving the way for more personalized and effective cancer management strategies. 

#### 2.1.2. Immunomagnetic Separation

Immunomagnetic separation has become a cornerstone in the isolation of circulating tumor cells (CTCs), leveraging the specificity of antigen–antibody interactions to enhance the precision of CTC capture. This method employs magnetic particles coated with antibodies that target specific markers on the surface of CTCs, enabling their separation from other blood components.

##### Development and Mechanism

The principle of immunomagnetic separation involves the use of magnetic beads conjugated with antibodies that specifically bind to antigens expressed on CTCs. Once bound, these CTC-magnetic bead complexes can be manipulated and isolated using a magnetic field [33]. This technique has been integrated into microfluidic platforms, enhancing its efficiency and applicability in clinical settings [33,34,35].

##### Advantages

One of the primary advantages of immunomagnetic separation is its high specificity. By targeting specific cell surface markers, such as the epithelial cell adhesion molecule (EpCAM), this method can selectively isolate CTCs from blood samples with minimal contamination from other cell types [27]. Additionally, the integration of immunomagnetic separation with microfluidic technology has led to the development of compact and automated systems, which are capable of processing blood samples with a high throughput and efficiency [27,33].

##### Limitations and Challenges

Despite its advantages, immunomagnetic separation faces several challenges. A significant limitation is the reliance on the expression of specific markers by CTCs. Tumor cells undergoing epithelial-to-mesenchymal transition (EMT) may downregulate these markers, leading to their evasion from detection [24,27]. Furthermore, the technique’s dependence on the antigen–antibody interaction can be affected by the heterogeneity of CTCs, potentially resulting in variable capture efficiencies [27].

##### Clinical Applications and Future Directions

Immunomagnetic separation has shown promise in various clinical applications, including the monitoring of disease progression, assessment of treatment response, and evaluation of metastatic potential. The method’s ability to provide the rapid and specific isolation of CTCs makes it a valuable tool in the realm of liquid biopsy [27,35]. Future developments are expected to focus on enhancing the sensitivity of this technique, expanding its applicability to a broader range of cancer types, and integrating it with other diagnostic modalities for comprehensive cancer analysis [27,32].

#### 2.1.3. Advanced Imaging and Analysis

The advancement in imaging technologies has significantly enhanced the detection and analysis of circulating tumor cells (CTCs), offering new insights into cancer metastasis and treatment response. While the real-time imaging of CTCs in human patients presents considerable challenges due to their rarity and transient nature, innovative approaches using multi-channel epifluorescence microscope imaging systems and model organisms like zebrafish have provided valuable platforms for studying CTC behavior and interactions, guiding their use in liquid biopsies.

##### Imaging Challenges and Alternative Models

The real-time imaging of CTCs in human patients is extremely challenging due to the technical limitations in tracking these rare cells as they circulate in the bloodstream. However, the use of zebrafish models has emerged as a powerful tool to overcome these challenges. Zebrafish are particularly advantageous due to their transparent embryos and rapid development, allowing for the detailed observation of tumor cell behavior in a living organism [36,37,38,39,40,41,42]. Studies have successfully utilized zebrafish models to observe the extravasation of CTCs, providing critical insights into the mechanisms of cancer cell migration and metastasis [43,44]. The information gleaned from these imaging techniques have shaped the understanding of findings traditional liquid biopsies allow for and advanced in the understanding of the clinical significance of cellular and molecular readouts.

##### Advancements in Imaging Techniques

One significant advancement is in the field of epifluorescence microscopy. The development of multi-channel epifluorescence microscope imaging systems is pivotal. These systems are designed to scan samples axially under multiple channels, capturing cell-specific biomarker expression. This approach allows for the creation of detailed, multi-color, all-in-focus whole slide images, crucial for an accurate analysis of CTCs. The integration of such advanced imaging techniques into the detection of CTCs enhances the sensitivity and specificity of CTC identification, which is essential for understanding the complex behavior of these cells in cancer progression [45,46].

Another area of advancement is in the field of auto-focusing technologies for slide scanners. The development of slide scanners with enhanced auto-focusing abilities addresses the challenge of acquiring uniform in-focus scans of microfilters used in CTC detection. These scanners employ a customized scanning strategy that mimics manual focusing, improving the accuracy and reliability of CTC detection. Such technological advancements are critical in clinical settings, where high accuracy and confidence in sample analysis are paramount [45,46,47].

Other recent advancements in imaging techniques, such as intravital microscopy and light-sheet fluorescence microscopy, have enabled the detailed visualization of CTCs in these model systems. These technologies allow for the high-resolution, three-dimensional imaging of CTCs, capturing their dynamic interactions with the vasculature and surrounding tissues. For instance, intravital microscopy has been used to study the process of *angiopellosis*, where CTCs actively remodel blood vessels to facilitate their extravasation [43].

##### Molecular Characterization and Analysis

Advanced imaging is often coupled with molecular characterization techniques, such as RNA sequencing, to analyze the gene expression profiles of CTCs and their clusters. This combination of imaging and molecular analysis has led to the identification of key pathways and genes involved in CTC extravasation and metastasis, such as the versican/extracellular matrix remodeling pathway in osteosarcoma [44]. These findings have significant implications for understanding the molecular drivers of metastasis and identifying potential therapeutic targets. Additionally, these techniques lay the groundwork for identifying key biomarkers that can be used in conjunction with liquid biopsies to determine the state and progression level of cancer in patients. 

##### Clinical Implications

Overall, these advancements in imaging technologies are instrumental in advancing our understanding of CTC dynamics. The enhanced imaging capabilities allow for a more detailed and accurate analysis of CTCs, which is essential for unraveling the complexities of cancer metastasis and guiding clinical decisions. The integration of these technologies into clinical practice can potentially transform cancer diagnosis and treatment, paving the way for more personalized and effective cancer care. Furthermore, the insights gained from imaging studies in model organisms like zebrafish and mice can inform the development of novel therapeutic strategies targeting the metastatic cascade [17,43,44].

### 2.2. Clinical Applications of CTCs

#### 2.2.1. Monitoring Treatment Response

The use of circulating tumor cells (CTCs) in monitoring cancer treatment response represents a significant advancement in personalized oncology. CTCs, as a component of liquid biopsies, offer a dynamic and non-invasive means to evaluate the effectiveness of therapeutic interventions in real time.

##### Role of CTCs in Treatment Monitoring

CTCs provide crucial insights into the tumor’s response to treatment. Changes in CTC counts and their molecular characteristics can indicate whether a cancer is responding to therapy, remaining stable, or progressing. For instance, a decrease in CTC levels during treatment often correlates with a positive response, while an increase can signal resistance or disease progression [48,49]. This real-time monitoring capability is particularly valuable in guiding treatment adjustments and in the early detection of metastasis [21,28,50,51].

##### Molecular Profiling of CTCs

Advanced molecular profiling techniques, such as next-generation sequencing (NGS) and single-cell analysis, have enabled the detailed characterization of CTCs. These methods can identify specific genetic mutations, expression patterns, and other biomarkers that are crucial for understanding the tumor’s response to specific drugs. For example, the detection of specific drug resistance mutations in CTCs can guide the selection of alternative therapies [6,22,52].

##### Clinical Applications

The clinical application of CTCs in treatment monitoring has been demonstrated in various cancer types. In breast cancer, for instance, CTC counts have been used to predict patient survival and assess the efficacy of chemotherapy [51,53]. In prostate cancer, the presence of CTCs has been linked to treatment resistance and disease progression [54,55,56,57].

##### Challenges and Future Directions

Despite their potential, the use of CTCs in monitoring treatment response faces challenges. The heterogeneity of CTCs and the complexity of their isolation and analysis require standardized protocols and sensitive detection methods. Future research is focused on improving the sensitivity and specificity of CTC detection and expanding their application across a broader range of cancer types [4].

Innovative research, such as that by Hayes et al. (2006) and Scher et al. (2009), underscores the clinical utility of CTC counts in monitoring treatment response and disease progression, offering a non-invasive method to guide therapeutic decisions and improve patient outcomes [53,57].

#### 2.2.2. Prognostic Value of Circulating Tumor Cell Counts

The prognostic significance of circulating tumor cell (CTC) counts in various cancers has become an area of intense research focus. CTCs, shed from primary or metastatic tumors into the bloodstream, serve as potential indicators of disease progression and patient prognosis.

##### Prognostic Implications across Cancer Types

In breast cancer, the presence and quantity of CTCs have been consistently linked to a poorer prognosis. Studies have shown that higher CTC counts correlate with reduced progression-free and overall survival [58]. Similarly, in colorectal cancer, CTC detection is associated with advanced disease stages and poorer survival outcomes [59]. In prostate cancer, CTC enumeration has been used to predict survival in metastatic cases, particularly in castration-resistant prostate cancer [57].

##### CTCs as Dynamic Prognostic Markers

CTC counts are not static; they can change in response to treatment or disease progression. This dynamic nature makes them valuable for monitoring disease status over time. For instance, an increase in CTC levels during treatment can indicate resistance or disease progression, while a decrease may suggest a favorable response to therapy [53].

##### Challenges in Utilizing CTC Counts

Despite their potential, the use of CTC counts as a prognostic tool faces challenges. Variability in CTC detection methods and the biological heterogeneity of CTCs can affect the accuracy and consistency of measurements. Additionally, the lack of standardized thresholds for CTC counts across different cancer types complicates their interpretation in clinical settings [50].

##### Future Perspectives

Ongoing research aims to standardize CTC enumeration techniques and integrate CTC analysis with other biomarkers for a more comprehensive prognostic assessment. Advances in single-cell sequencing and the molecular characterization of CTCs are expected to provide deeper insights into their prognostic significance and may lead to more personalized therapeutic strategies [6,23,54,60,61].

#### 2.2.3. Personalized Therapy: The Role of CTC Analysis

Circulating tumor cell (CTC) analysis has emerged as a transformative approach in the realm of personalized therapy for cancer patients. By providing a real-time snapshot of tumor characteristics, CTCs offer invaluable insights that can guide the development of individualized treatment plans.

##### Tailoring Treatment Based on CTC Characteristics

CTC analysis allows for the identification of specific genetic mutations, protein expressions, and other biomarkers that are crucial in determining the most effective treatment for a particular patient. For instance, in breast cancer, the presence of HER2-positive CTCs might prompt the use of HER2-targeted therapies, even if the primary tumor is HER2-negative [62]. Similarly, in lung cancer, the detection of EGFR mutations in CTCs can guide the use of EGFR inhibitors [63].

##### Monitoring Drug Resistance and Disease Evolution

CTCs can also provide insights into the development of drug resistance. By analyzing changes in CTCs over time, clinicians can identify resistance mechanisms, such as the emergence of new mutations or phenotypic changes, enabling them to adjust treatment strategies accordingly [64]. This aspect is particularly crucial in managing diseases like metastatic prostate cancer, where CTCs can reveal evolving resistance to androgen-deprivation therapy [65].

##### Challenges in CTC-Based Personalized Therapy

Despite its potential, the application of CTC analysis in personalized therapy faces several challenges. The heterogeneity of CTCs, coupled with the technical difficulties in isolating and analyzing these rare cells, can complicate their use in clinical settings. Moreover, the interpretation of CTC data requires a deep understanding of tumor biology and the complex interactions between tumor cells, their microenvironment, and extracellular vesicles [4,66].

##### Future Directions

Advancements in CTC isolation techniques and molecular analysis are expected to enhance the utility of CTCs in personalized therapy. The integration of CTC analysis with other liquid biopsy components, such as cell-free DNA (cfDNA) [67], is anticipated to provide a more comprehensive view of tumor genetics and dynamics. Ongoing research is focused on developing more sensitive and specific methods for CTC detection and characterization, paving the way for more effective and tailored cancer treatments [52].

### 2.3. CTCs vs. Traditional Biopsy Methods

#### 2.3.1. Invasiveness and Patient Comfort

The comparison between circulating tumor cell (CTC) analysis and traditional biopsy methods in terms of invasiveness and patient comfort is crucial in understanding the advancements in cancer diagnostics and patient care [50].

##### Invasiveness of Traditional Biopsies

Traditional biopsies, often considered the gold standard for cancer diagnosis, involve the physical extraction of tissue from the tumor site. This procedure can be invasive, at times requiring surgical intervention, which may lead to complications such as pain, bleeding, infection, and, in some cases, significant recovery time [68]. The invasiveness of traditional biopsies can be particularly challenging for patients with tumors in hard-to-reach or sensitive areas, and for those who require multiple biopsies over time to monitor disease progression or treatment response (Table 3) [52].

**Table 2:** Emerging technologies for detection and isolation of CTCs.

Technology	Description	Advantages	Disadvantages	References
Acoustic Separation Methods	Utilizes sound waves to isolate CTCs based on size and physical properties.	Non-contact and label-free isolation, preserving cell integrity and viability; suitable for a wide range of cell types.	May require specialized equipment and expertise; potential limitations in throughput.	Bhat, M.P., et al. (2022). “Recent Advances in Microfluidic Platform for Physical and Immunological Detection and Capture of Circulating Tumor Cells.” Biosensors, 12(4), 220 [30]
Parsortix System by Angle	A microfluidic device that captures and enumerates live CTCs based on size exclusion.	Allows for the capture and enumeration of live CTCs; can be used for downstream analyses.	May not capture CTCs that do not express the targeted markers; dependency on device availability.	Farhang Ghahremani, M., et al. (2023). “Novel method for highly multiplexed gene expression profiling of circulating tumor cells (CTCs) captured from the blood of women with metastatic breast cancer.” Journal of Translational Medicine, 21, 414 [69]
Rarecyte’s CyteFinder II	An imaging platform that enables enumeration and analysis of CTCs using multiplexed immunofluorescence.	High specificity and sensitivity in CTC detection; enables detailed cellular analysis.	Requires high-quality antibodies for specific detection; potentially high operational costs.	Takagi, H., et al. (2020). “Analysis of the Circulating Tumor Cell Capture Ability of a Slit Filter-Based Method in Comparison to a Selection-Free Method in Multiple Cancer Types.” International Journal of Molecular Sciences, 21(23), 9031 [70]
CTCelect by ScreenCell	A size-based filtration device designed to isolate circulating tumor cells (CTCs) from blood samples.	Simplifies the CTC isolation process; maintains high efficiency and cell viability for downstream analyses; does not require cell-surface-marker-based selection, allowing for the capture of a broader range of CTC phenotypes.	Size-based selection may miss smaller CTCs or capture non-tumor cells of similar size; potential for clogging with high cell count samples.	Stiefel, J., et al. (2022). “Characterization of a novel microfluidic platform for the isolation of rare single cells to enable CTC analysis from head and neck squamous cell carcinoma patients.” Engineering in Life Sciences, 22(5), 391–406 [29]

**Table 3:** Comparison of CTC liquid biopsy vs. traditional biopsy methods.

	CTC Liquid Biopsy	Traditional Biopsy
Invasiveness	Low invasiveness	High invasiveness
Patient comfort	Minimal patient discomfort (blood draw)	Potential high discomfort (surgical procedure)
Predicting therapeutic response	Changes in levels can predict response/resistance/relapse	Changes in levels predict response/resistance/relapse
Ability to assess genomic/transcriptomic/protein data	Can analyze DNA, RNA, and protein	Can analyze DNA, RNA, and protein
Diagnostic accuracy	Potential discrepancies in CTC detection due to varying expression of surface markers and heterogeneity	Comprehensive information about the tumor architecture and microenvironment
Tumor representativeness	Representing the current state of the tumor, including its metastatic potential	Provide a snapshot of the tumor at the time of biopsy
Single-cell examination	Can analyze CTCs at the single-cell resolution in circulation	Can analyze CTCs at the single-cell resolution at primary site
Challenges in collection/interpretation	Heterogeneity in CTCs can affect analysis	May not always reflect the current status of the tumor, especially in cases of metastatic or rapidly evolving cancers
	Sampling bias of captured cells (high affinity and larger size)	

##### Minimally Invasive Nature of CTC Analysis

In contrast, CTC analysis through liquid biopsies is a minimally invasive procedure that involves a simple blood draw, similar to a routine blood test [23]. This liquid biopsy technique reduces the physical discomfort and risk associated with traditional biopsies. The non-invasive nature of CTC analysis is particularly advantageous for patients who are not candidates for surgical biopsy due to their medical condition or the location of the tumor [16]. 

##### Considerations in Diagnostic Accuracy

While CTC analysis offers advantages in terms of invasiveness and patient comfort, it is important to consider that, in some cases, traditional biopsies may provide more comprehensive information about the tumor, such as its architecture and microenvironment [6,48,58]. However, advancements in CTC analysis techniques are continually improving the diagnostic and prognostic value of this method [19].

##### Conclusions

CTC analysis and other liquid biopsy techniques are a significant advancement in cancer diagnostics, offering a less invasive and more patient-friendly alternative to traditional biopsies [71]. As technology progresses, it is likely that CTC analysis will play an increasingly important role in cancer diagnosis, treatment monitoring, and personalized medicine, complementing traditional biopsy methods.

#### 2.3.2. Accuracy and Representativeness: CTCs vs. Tissue Biopsies

The comparison of circulating tumor cells (CTCs) and traditional tissue biopsies in terms of accuracy and tumor representativeness is crucial in evaluating their respective roles in cancer diagnosis and treatment planning.

##### Accuracy of CTC Analysis

CTC analysis has shown promise in accurately detecting and characterizing tumor cells in the bloodstream. Advances in isolation and detection technologies have improved the sensitivity and specificity of CTC analysis [50]. However, the accuracy can be influenced by the heterogeneity of CTCs and the varying expression of surface markers used for their isolation. This variability can sometimes lead to discrepancies in CTC detection, particularly in cases where tumor cells undergo phenotypic changes, such as epithelial-to-mesenchymal transition (EMT) [16].

##### Tumor Representativeness of CTCs

CTCs offer a unique advantage in representing the current state of the tumor, including its metastatic potential. They can provide real-time insights into tumor dynamics and molecular changes, which are crucial for monitoring disease progression and treatment response [52]. However, one limitation is that CTCs may not always capture the full genomic landscape of the primary tumor, especially in heterogeneous tumors where different subclones may be present [23]. 

##### Representativeness of Tissue Biopsies

Traditional tissue biopsies provide a snapshot of the tumor at the time of biopsy. They offer detailed histological and molecular information about the tumor, which is essential for the initial diagnosis and treatment planning. Tissue biopsies are particularly valuable in analyzing the tumor microenvironment and its interactions with the tumor cells [68]. However, they may not always reflect the current status of the tumor, especially in cases of metastatic or rapidly evolving cancers [9,28,72,73].

##### Challenges in Comparing CTCs and Tissue Biopsies

Comparing the accuracy and representativeness of CTCs and tissue biopsies is challenging due to the inherent differences in their nature. CTCs provide a dynamic and systemic view of the tumor, while tissue biopsies offer a localized but detailed snapshot. The choice between these methods depends on various factors, including the cancer type, stage, and clinical objectives [8,49,74,75].

##### Conclusions

Both CTC analysis and tissue biopsies have their strengths and limitations in terms of accuracy and tumor representativeness. The integration of both methods can provide a more comprehensive understanding of the tumor, guiding more effective and personalized treatment strategies.

Comparative analyses by Lin et al. (2021) and Crowley et al. (2013) have highlighted the less invasive nature and potential for real-time disease monitoring offered by CTC analysis, challenging the traditional reliance on tissue biopsies and paving the way for more patient-friendly diagnostic approaches [6,48].

### 2.4. Emergence of CTC Clusters as Fundamental Targets for Liquid Biopsies

#### 2.4.1. Significance of CTC Clusters in Metastasis and Prognosis

Circulating tumor cell (CTC) clusters have increasingly been recognized as pivotal elements in cancer metastasis. Contrasting with individual CTCs, these multicellular aggregates exhibit a heightened potential for metastasis [10,76]. The presence of CTC clusters is not only associated with increased metastatic potential but also correlates with higher mortality rates, enhanced treatment resistance, and a poorer prognosis overall [76,77,78]. These findings underscore the critical role CTC clusters play in the progression of various cancers.

#### 2.4.2. Origin and Metastatic Journey of CTC Clusters

Recent breakthroughs have shifted the paradigm regarding the origin and behavior of CTC clusters. Earlier theories posited that these clusters formed from individual CTCs that amalgamate during circulation. However, new evidence also suggests that CTC clusters can detach from the primary tumor as pre-formed groups, maintaining their multicellular structure during transit in the bloodstream [9,79,80]. Furthermore, contrary to previous beliefs that CTC clusters must disband into single cells to seed secondary tumors, it has been discovered that these clusters can extravasate as intact groups [43,44,81]. This process, known as *angiopellosis*, allows them to retain their multicellular phenotype and effectively proliferate at distant sites, a phenomenon central to the *Cancer Exodus Hypothesis* [11,76,77,81,82,83]. Advanced imaging and isolation techniques have been instrumental in observing these processes, revealing that CTC clusters exhibit unique molecular profiles that enhance their metastatic capabilities [13,31,83,84,85,86].

Research by Aceto et al. (2014) and Amintas et al. (2020) has brought to light the enhanced metastatic potential of CTC clusters, offering new insights into the mechanisms of cancer spread and identifying them as crucial targets for therapeutic intervention [9,10].

#### 2.4.3. Advancements in Liquid Biopsy Targeting CTC Clusters

The evolving field of liquid biopsy has significantly benefited from these insights into CTC clusters. Progress in microfluidics and other isolation technologies has made it feasible to selectively isolate CTCs as either individual cells or clusters [31,44,83,87,88]. This advancement is crucial for making clinically relevant assessments about cancer progression and guiding treatment strategies. The ability to target CTC clusters in liquid biopsies represents a substantial leap forward in personalized medicine and cancer management [89].

#### 2.4.4. Future Directions in CTC Cluster Research

The focus on CTC clusters is paramount, given their substantial role in metastasis, the primary cause of cancer mortality. Future research in this area is vital for developing more effective diagnostic and therapeutic approaches. By understanding the mechanisms through which CTC clusters contribute to cancer spread, new strategies can be devised to intercept these processes, potentially reducing the incidence of metastasis and improving patient outcomes [76,77,89,90].

The collective insights into the behavior, origins, and clinical implications of CTC clusters not only enrich our understanding of cancer biology but also open new avenues in the fight against this disease. Emphasizing CTC clusters in research and clinical practice could lead to significant advancements in cancer diagnostics and therapeutics.

### 2.5. Challenges and Limitations

#### 2.5.1. Detection Sensitivity and Specificity of CTCs

The detection of circulating tumor cells (CTCs) presents significant challenges in terms of sensitivity and specificity, which are critical for their effective use in cancer diagnosis and management.

##### Sensitivity Challenges in CTC Detection

One of the primary challenges in CTC detection is sensitivity. CTCs are often present in very low numbers in the bloodstream, especially in the early stages of cancer or in non-metastatic cases. The rarity of these cells necessitates highly sensitive detection methods to accurately identify and enumerate them [4]. Current technologies, while advancing, sometimes fail to detect CTCs in all patients with known metastatic disease, leading to false-negative results. This limitation can impact the clinical utility of CTC analysis, particularly in early cancer detection and in monitoring minimal residual disease [23].

##### Specificity Issues in CTC Identification

Specificity in CTC detection is another challenge. It involves the ability to distinguish CTCs from other non-tumor cells circulating in the blood, such as blood cells or benign epithelial cells. The heterogeneity of CTCs, in terms of size, shape, and marker expression, adds complexity to this task. Some CTC isolation techniques rely on surface markers, like EpCAM, which may not be expressed on all CTCs, particularly those that have undergone epithelial-to-mesenchymal transition (EMT), a process associated with increased metastatic potential [17].

##### Technological Advances and Limitations

Advances in microfluidics, immunomagnetic separation, and imaging have improved the sensitivity and specificity of CTC detection. However, these technologies still face limitations in terms of throughput, efficiency, and the ability to provide a comprehensive characterization of CTCs. The balance between high sensitivity (to capture as many CTCs as possible) and high specificity (to ensure the captured cells are indeed CTCs) remains a critical area of ongoing research [52].

##### Conclusions

The challenges in the sensitivity and specificity of CTC detection represent significant hurdles in the clinical application of CTC analysis. Addressing these challenges is crucial for improving the reliability of CTCs as biomarkers in cancer diagnosis, prognosis, and treatment monitoring. Continued technological advancements and research are essential to overcome these limitations and fully harness the potential of CTCs in clinical oncology.

#### 2.5.2. Standardization and Clinical Validation: CTC-Based Diagnostics

The implementation of circulating tumor cell (CTC) analysis in clinical practice necessitates standardized protocols and rigorous clinical validation. These aspects are crucial for ensuring the reliability, reproducibility, and clinical utility of CTC-based diagnostics.

##### The Need for Standardized Protocols

Currently, one of the major challenges in CTC research and clinical application is the lack of standardized methods for CTC isolation and analysis. Different techniques, such as immunomagnetic separation, microfluidics, and filtration-based methods, vary in their efficiency, sensitivity, and specificity [7,26,33,50,59,91]. This variability can lead to inconsistent results across different laboratories and studies, making it difficult to compare and interpret findings. The standardization of protocols, including sample collection, CTC isolation, and analysis, is essential in order to achieve consistent and reliable results that can be universally accepted and applied in clinical settings [4].

##### Clinical Validation of CTC-Based Diagnostics

Although diagnostics from research involving liquid biopsies of CTCs have been developed, many still remain unvalidated and their clinical significance is not fully understood. The clinical validation of CTC-based diagnostics involves demonstrating their accuracy, reliability, and clinical relevance through extensive clinical trials and studies. This process is crucial to establish the prognostic and predictive value of CTCs in various cancer types and stages. Clinical validation also includes assessing the utility of CTCs in monitoring treatment response, detecting minimal residual disease, and guiding personalized therapy decisions [49,91].

##### Regulatory Considerations

The integration of CTC-based diagnostics into routine clinical practice also involves regulatory considerations. Diagnostic tests must meet regulatory standards and obtain approval from bodies such as the U.S. Food and Drug Administration (FDA) or the European Medicines Agency (EMA). This process ensures that the tests are safe, effective, and provide meaningful clinical information [1,23,92,93].

##### Future Directions

Efforts are ongoing to develop and validate standardized CTC assays. Collaborative initiatives among researchers, clinicians, and regulatory agencies are essential for establishing guidelines and consensus on the best practices for CTC analysis. The future of CTC-based diagnostics lies in their integration into personalized medicine, where they can provide real-time insights into tumor biology and treatment efficacy, ultimately improving patient outcomes [19,21,49,50].

### 2.6. The Next Frontier of CTC Liquid Biopsies

#### 2.6.1. Non-CTC Liquid Biopsy Technologies

Building upon the foundational understanding of liquid biopsy and the pivotal role of circulating tumor cells (CTCs) discussed earlier, it is essential to delve deeper into the other critical components of this innovative diagnostic approach: cell-free DNA (cfDNA)/RNA and exosomes. These biomarkers complement CTCs by providing additional layers of insight into the tumor’s genetic and molecular characteristics, each with their unique advantages for clinical application [94].

##### Circulating Tumor Cells (CTCs) vs. Cell-Free DNA/RNA

CTCs provide a direct representation of tumor cells circulating in the bloodstream, offering real-time information on tumor dynamics and the potential for metastasis. Their analysis can reveal critical information on tumor heterogeneity, drug resistance mechanisms, and metastatic potential. However, the rarity and heterogeneity of CTCs pose significant challenges for their isolation and analysis, requiring highly sensitive and specific technologies.

In contrast, cell-free DNA (cfDNA) and RNA (cfRNA) analysis involves the detection of DNA and RNA fragments released into the bloodstream from tumor cells. cfDNA/RNA offers advantages in terms of abundance and ease of detection, allowing for the identification of genetic mutations, copy number alterations, and gene expression profiles associated with the tumor. However, cfDNA/RNA lacks the cellular context provided by CTCs, which can be crucial for understanding the phenotypic characteristics of the tumor cells and their microenvironment interactions [95].

##### CTCs vs. Exosomes

Exosomes are small extracellular vesicles released by cells, including tumor cells, into the bloodstream. They carry a wide range of biomolecules, including DNA, RNA, proteins, and lipids, reflective of their cell of origin. Exosome analysis can provide a comprehensive molecular profiling of the tumor, offering insights into the signaling pathways and tumor microenvironment. The non-invasive nature of exosome isolation and the stability of their cargo make them an attractive option for liquid biopsy [96,97].

However, similar to cfDNA/RNA, exosomes do not provide information on the physical characteristics of tumor cells, such as morphology and cell surface markers, which can be critical for certain diagnostic and prognostic applications. Moreover, distinguishing tumor-derived exosomes from those originating from normal cells remains a technical challenge.

##### Conclusions

The choice between CTCs, cfDNA/RNA, and exosomes for liquid biopsy applications depends on the specific clinical question being addressed. CTC analysis offers unparalleled insights into the cellular aspects of the tumor, cfDNA/RNA provides a broad snapshot of the genetic alterations, and exosomes offer a holistic view of the tumor’s molecular makeup. A comprehensive liquid biopsy approach that integrates the analysis of CTCs, cfDNA/RNA, and exosomes can provide the most complete picture of the tumor, enhancing the precision of cancer diagnosis, prognosis, and monitoring [98,99].

#### 2.6.2. Integration with Other Biomarkers

The integration of circulating tumor cell (CTC) analysis with other biomarkers represents a promising direction in cancer diagnostics and treatment. This approach aims to provide a more comprehensive view of cancer biology, enhancing the accuracy and effectiveness of cancer profiling.

##### Combining CTC Analysis with Other Liquid Biopsy Markers

CTC analysis, when combined with the analysis of other liquid biopsy markers such as cell-free DNA (cfDNA), circulating tumor RNA (ctRNA), and exosomes, can offer a more complete picture of the tumor’s genetic and molecular landscape. For instance, cfDNA can provide information on genetic mutations and alterations that are not always detectable in CTCs [52,67]. Similarly, ctRNA and exosomes can offer insights into the gene expression profiles and signaling pathways active in tumor cells [100,101]. Another area of interest is the development of methods to detect markers on CTCs that may predict the response to immunotherapy [102,103,104].

##### Enhancing Precision Medicine in Oncology

The integration of multiple biomarkers can significantly enhance the precision of personalized medicine. By analyzing a combination of biomarkers, clinicians can gain a deeper understanding of tumor heterogeneity, monitor disease progression more accurately, and tailor treatments to target specific molecular characteristics of the tumor [6]. This approach is particularly valuable in tracking the evolution of tumors and identifying emerging resistance mechanisms to targeted therapies [16].

##### Challenges and Opportunities

The integration of CTC analysis with other biomarkers presents both challenges and opportunities. One challenge lies in developing and standardizing methods to simultaneously isolate and analyze different types of biomarkers from a single blood sample. Additionally, interpreting the complex data generated from multi-marker analysis requires advanced bioinformatics tools and expertise [4,105].

##### Future Research and Clinical Applications

Future research in this area is likely to focus on refining techniques for simultaneous biomarker analysis and developing integrated platforms that can provide comprehensive cancer profiling from liquid biopsies. Clinical trials are also needed to validate the clinical utility of multi-marker analysis in guiding treatment decisions and improving patient outcomes [19,49,51,87].

#### 2.6.3. Technological Innovations

The future of circulating tumor cell (CTC) detection and analysis is marked by promising technological advancements, including the integration of artificial intelligence (AI) and machine learning, as well as other innovative techniques. These developments are set to significantly enhance the precision and utility of CTC analysis in cancer care.

##### AI and Machine Learning in CTC Analysis

AI and machine learning are poised to revolutionize CTC analysis by enabling the processing of complex data sets to identify patterns and biomarkers. These technologies can improve the accuracy of CTC detection, characterize their morphological and genetic features, and even predict treatment responses and patient outcomes more effectively [106,107,108,109,110]. AI algorithms can process and analyze vast amounts of data from CTCs, identifying patterns and biomarkers that might be missed by traditional methods. Machine-learning models can be trained to recognize specific CTC characteristics, such as morphological features, genetic mutations, and protein expressions, enhancing the accuracy and efficiency of CTC detection and characterization [85,106,109].

Additionally, AI-driven analysis models have the potential to provide deeper insights into cancer biology. They can analyze CTCs in conjunction with other biomarkers, such as cell-free DNA (cfDNA) and RNA, to create comprehensive profiles of tumors. This approach can lead to more accurate predictions of disease progression, treatment response, and patient outcomes. Machine-learning models can also adapt and improve over time, learning from new data to refine their predictive capabilities [105,111,112,113].

##### Microfluidics and Nanotechnology

Advancements in microfluidics and nanotechnology are also transforming CTC detection. Microfluidic devices allow for the precise manipulation of fluids at a micro-scale, improving the isolation and capture of CTCs. Nanotechnology, particularly the use of nanoparticles, can enhance the sensitivity of CTC detection by targeting specific tumor markers or properties [13,25,26,31,34,85].

##### Single-Cell Analysis

Single-cell analysis technologies are advancing rapidly, offering detailed insights into the heterogeneity of CTCs. This approach allows for the examination of individual CTCs, providing a deeper understanding of their molecular characteristics and the tumor’s genetic diversity. This information is crucial for personalized treatment strategies and understanding drug resistance mechanisms [90,114].

##### Integration with Genomic and Proteomic Analysis

The integration of CTC analysis with genomic and proteomic profiling is another area of significant development. This comprehensive approach can provide a more complete picture of the tumor’s molecular landscape, aiding in the identification of actionable mutations and novel therapeutic targets [88,115,116].

##### Challenges and Future Research

While these technological advancements hold great promise, challenges remain in standardizing these methods and ensuring their clinical applicability. Future research will focus on validating these technologies in clinical trials, ensuring their accuracy, and integrating them into routine clinical practice for personalized cancer management [4].

The future of CTC detection and analysis is bright, with AI and machine learning, microfluidics, nanotechnology, single-cell analysis, and genomic and proteomic integration leading the way. These advancements are expected to transform cancer diagnostics and treatment, paving the way for more precise, efficient, and personalized oncology care.

## 3. Conclusions

The exploration of CTCs as a cornerstone of liquid biopsy represents a transformative leap in the field of oncology. This review has underscored the multifaceted role of CTCs in advancing our understanding of cancer biology, particularly in the context of metastasis, treatment response, and personalized therapy. The technological advancements in CTC detection and isolation, including microfluidics, immunomagnetic separation, and advanced imaging, have not only enhanced the sensitivity and specificity of CTC analysis, but also opened new avenues for their characterization. These developments have paved the way for the real-time monitoring of cancer progression, offering a window into tumor dynamics and heterogeneity that was previously unattainable with traditional biopsy methods.

Advancements in the detection, enumeration, and characterization of CTCs has the potential to contribute significantly to our understanding and improved treatment of cancer. From the early detection and monitoring of minimal residual disease to the assessment of therapeutic efficacy and the development of personalized treatment plans, CTCs are at the forefront of a new era in cancer care. The integration of CTC analysis with other liquid biopsy components, such as cell-free DNA and RNA, is anticipated to provide a more comprehensive view of tumor genetics and dynamics. This holistic approach is expected to guide more effective and tailored cancer treatments, ultimately improving patient outcomes.

However, challenges remain in the standardization and clinical validation of CTC-based diagnostics. Addressing these challenges is crucial for improving the reliability of CTCs as biomarkers in cancer diagnosis, prognosis, and treatment monitoring. Continued technological advancements and research are essential for overcoming these limitations and fully harnessing the potential of CTCs in clinical oncology.

Looking forward, the integration of artificial intelligence and machine learning in CTC analysis, coupled with advancements in microfluidics, nanotechnology, and single-cell analysis, promises to revolutionize cancer diagnostics and treatment. These innovations are expected to enhance the precision, efficiency, and personalization of oncology care, marking a new chapter in the battle against cancer.

In conclusion, CTCs represent a pivotal element in the evolving landscape of cancer management. Their potential to transform cancer diagnosis, prognosis, and therapy is immense, heralding a future where cancer treatment is more dynamic, precise, and patient-centric. As we continue to unravel the complexities of CTC biology and refine their clinical applications, the promise of improved cancer care and patient outcomes becomes increasingly tangible.

## Figures and Tables

**Table 1:** Established Technologies for detection and isolation of CTCs.

Technology	Description	Advantages	Disadvantages
Immunomagnetic Separation	Utilizes magnetic particles coated with antibodies to isolate CTCs based on specific antigen–antibody interactions.	High specificity, can be integrated into microfluidic systems.	May miss CTCs with low expression of targeted markers due to epithelial-to-mesenchymal transition.
Microfluidics	Employs devices that manipulate fluids on a micro-scale to isolate CTCs based on physical properties such as size and deformability.	Allows for the integration of various functions (e.g., sorting and analysis), high-throughput processing.	Requires precise control of fluid dynamics and sophisticated device fabrication.
Digital PCR and BEAMing PCR	Highly sensitive methods for detecting specific mutations in CTCs, using digital partitioning of samples and PCR amplification.	High sensitivity for mutation detection, suitable for analyzing minimal quantities of DNA.	Focuses on predefined genetic alterations, may not capture the full heterogeneity of CTCs.

## Data Availability

No new data were created in this review article.
